# Projecting hospital utilization during the COVID-19 outbreaks in the United States

**DOI:** 10.1073/pnas.2004064117

**Published:** 2020-04-03

**Authors:** Seyed M. Moghadas, Affan Shoukat, Meagan C. Fitzpatrick, Chad R. Wells, Pratha Sah, Abhishek Pandey, Jeffrey D. Sachs, Zheng Wang, Lauren A. Meyers, Burton H. Singer, Alison P. Galvani

**Affiliations:** ^a^Agent-Based Modelling Laboratory, York University, Toronto, ON M3J 1P3, Canada;; ^b^Center for Infectious Disease Modeling and Analysis, Yale School of Public Health, New Haven, CT 06510;; ^c^Center for Vaccine Development and Global Health, University of Maryland School of Medicine, Baltimore, MD 21201;; ^d^Center for Sustainable Development at Columbia University, Columbia University, New York, NY 10032;; ^e^Department of Biostatistics, Yale School of Public Health, New Haven, CT 06510;; ^f^Department of Integrative Biology, The University of Texas at Austin, Austin, TX 78712;; ^g^Emerging Pathogens Institute, University of Florida, Gainesville, FL 32610

**Keywords:** SARS–CoV-2, hospitalization, self-isolation, critical care need

## Abstract

In the wake of community coronavirus disease 2019 (COVID-19) transmission in the United States, there is a growing public health concern regarding the adequacy of resources to treat infected cases. Hospital beds, intensive care units (ICUs), and ventilators are vital for the treatment of patients with severe illness. To project the timing of the outbreak peak and the number of ICU beds required at peak, we simulated a COVID-19 outbreak parameterized with the US population demographics. In scenario analyses, we varied the delay from symptom onset to self-isolation, the proportion of symptomatic individuals practicing self-isolation, and the basic reproduction number *R*_0_. Without self-isolation, when *R*_0_ = 2.5, treatment of critically ill individuals at the outbreak peak would require 3.8 times more ICU beds than exist in the United States. Self-isolation by 20% of cases 24 h after symptom onset would delay and flatten the outbreak trajectory, reducing the number of ICU beds needed at the peak by 48.4% (interquartile range 46.4–50.3%), although still exceeding existing capacity. When *R*_0_ = 2, twice as many ICU beds would be required at the peak of outbreak in the absence of self-isolation. In this scenario, the proportional impact of self-isolation within 24 h on reducing the peak number of ICU beds is substantially higher at 73.5% (interquartile range 71.4–75.3%). Our estimates underscore the inadequacy of critical care capacity to handle the burgeoning outbreak. Policies that encourage self-isolation, such as paid sick leave, may delay the epidemic peak, giving a window of time that could facilitate emergency mobilization to expand hospital capacity.

## Significance

Our results highlight that the growing coronavirus disease 2019 (COVID-19) outbreak in the United States could gravely challenge the critical care capacity, thereby exacerbating case fatality rates. In the absence of a preventive vaccine, efforts to contain the outbreak, such as improving self-isolation rates and encouraging better hygiene practices, can alleviate some of the pressures faced by the healthcare system during an outbreak. Both emergency expansion of hospital facilities to treat COVID-19 and government appropriations to facilitate voluntary case isolation are urgently needed.

The novel coronavirus (severe acute respiratory syndrome [SARS]–CoV-2) was first identified in December 2019 from a cluster of patients with severe pneumonia-like symptoms in Wuhan, China (1). As of March 11, 2020, the pandemic (coronavirus disease 2019, COVID-19) has already caused more than 135,000 confirmed cases across 117 countries (2). Despite travel restrictions, border control, and quarantine measures in China that delayed spread worldwide, the first case of COVID-19 in the United States was confirmed on January 20, 2020, arriving via an international flight from China on January 15 (3, 4). As of March 31, 2020, over 160,000 additional cases, the majority of which have arisen from local transmission, have been reported in several US states (5), indicating disseminated community spread of SARS–CoV-2 in the country.

Demand for critical care, including hospital beds and intensive care units (ICU), is expected to increase with the rising number of cases within the United States. An estimated 792,417 hospital beds are available in the United States, of which 97,776 are within ICUs (6). These resources are limited and usually function at more than half capacity outside public health emergencies (7). Previous estimates indicate that 65% of hospital beds (7) and ICU beds (8) are routinely occupied, absent a public health emergency. This translates to 277,346 typically unoccupied hospital beds and 34,222 unoccupied ICU beds. COVID-19 could overwhelm this limited resource. Estimation of potential coronavirus-driven demand for hospital and ICU beds is critical to inform operations dedicated to scaling up healthcare capacity.

Efforts are being implemented by the Centers for Disease Control and Prevention (CDC) and local health departments to ascertain optimal response strategies to mitigate the outbreak. In this study, we projected the COVID-19–associated demand for hospital and ICU beds within the United States. To this end, we developed an age-structured dynamic model of SARS–CoV-2 transmission, parameterized with the US population demographics and latest estimates from global COVID-19 outbreaks (*SI Appendix*, Table A5). We simulated disease spread under a range of scenarios for self-isolation, whereby symptomatic individuals reduce their contacts within the community by staying at home. Our results indicate that the COVID-19 outbreak will most likely overwhelm current hospital capacity and that expanding the number of ICU beds is an urgent imperative.

## Results

**Fig. 1.**
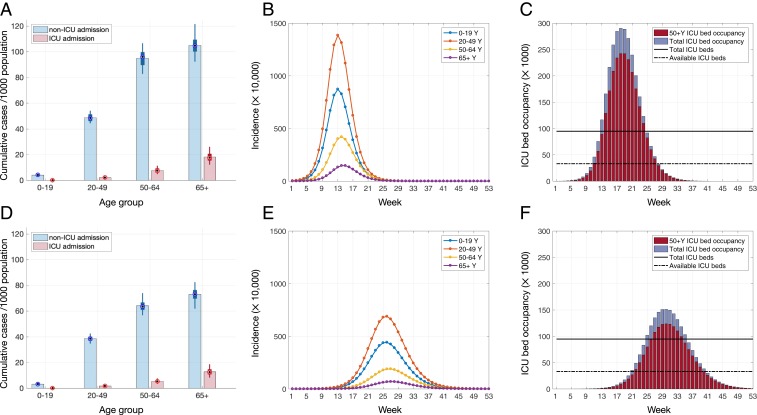
Projected outcomes for *R*_0_ = 2.5. (*A* and *D*) Rates of hospitalization and ICU admission in different age groups per 1,000 population. (*B* and *E*) Incidence of disease for different age groups. (*C* and *F*) Temporal ICU bed occupancy. Average time to self-isolation and proportion of individuals with mild symptoms practicing self-isolation are, respectively, (*A*–*C*) no self-isolation and (*D*–*F*) 24 h, 20%. Color bars in *A* and *D* illustrate the mean values, and box plots indicate the median and IQR of estimates. Solid and dashed lines in *C* and *F* indicate, respectively, the total ICU beds and availability based on reported occupancy rate of 65%.

**Fig. 2.**
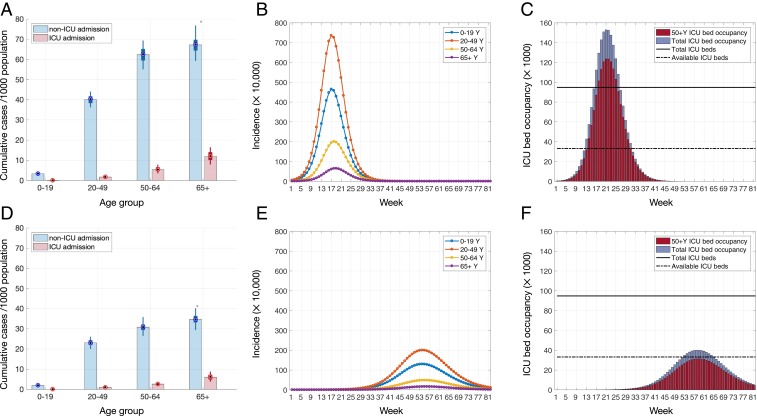
Projected outcomes for *R*_0_ = 2. (*A* and *D*) Rates of hospitalization and ICU admission in different age groups per 1,000 population. (*B* and *E*) Incidence of disease for different age groups. (*C* and *F*) Temporal ICU bed occupancy. Average time to self-isolation and proportion of individuals with mild symptoms practicing self-isolation are, respectively, (*A*–*C*) no self-isolation and (*D*–*F*) 24 h, 20%. Color bars in *A* and *D* illustrate the mean values, and box plots indicate the median and IQR of estimates. Solid and dashed lines in *C* and *F* indicate, respectively, the total ICU beds and availability based on reported occupancy rate of 65%.

We calibrated the transmission model to initial estimates (9) of the SARS–CoV-2 reproduction number *R*_0_ (i.e., the average number of secondary cases generated by a primary case) for the base case scenario *R*_0_ = 2.5, as well as the alternative scenario *R*_0_ = 2. In the absence of self-isolation, the cumulative incidence of infection was estimated to be 177 and 232 million for *R*_0_ = 2 and *R*_0_ = 2.5, respectively, which correspond to attack rates of ∼52% and 69%. Age-specific projections for hospitalization and ICU admission were also sensitive to the reproduction number (Figs. 1*A* and 2*A*). Among individuals over 50 y of age, we predicted 17.4 and 25.7 ICU admissions per 1,000 population for *R*_0_ = 2 and *R*_0_ = 2.5, respectively.

When *R*_0_ = 2.5, we projected that 293,520 (interquartile rage [IQR] 257,800 to 336,320) ICU beds would be required to treat critically ill cases at the outbreak peak, corresponding to 3.0 times more than the number of all existing ICU beds in the United States (Fig. 1*C*). For *R*_0_ = 2, the required number of ICU beds at the outbreak peak would be 150,940 (IQR 128,180 to 179,660), which is still 1.5 times higher than the number of all existing ICU beds (Fig. 2*C*). Accounting for a routine occupancy rate of 65% of ICU beds (7), the treatment of critically ill cases at the outbreak peak would require at least 8.6 and 4.4 times more ICU beds than typically unoccupied for *R*_0_ = 2.5 and *R*_0_ = 2, respectively (Figs. 1*C* and 2*C*). The need for total hospital beds at outbreak peak would be 3,034,676 (IQR 2,853,176 to 3,386,304) and 1,587,158 (IQR 1,450,974 to 1,798,820) for *R*_0_ = 2.5 and *R*_0_ = 2, respectively, equivalent to 10.9 and 5.8 times more than typically unoccupied.

Given that individuals are encouraged to self-isolate once symptomatic, we evaluated the extent to which this practice may mitigate overall rates of hospitalization and ICU admission, as well as the strain on hospital capacity at the outbreak peak. For *R*_0_ = 2.5, if delayed self-isolation were practiced by 5% of mildly symptomatic individuals 48 h after symptom onset, we projected 27.7 ICU admissions per 1,000 population, a decline of only 0.8% relative to no self-isolation. The weekly requirement for ICU beds at outbreak peak would reduce to 284,688 (IQR 236,860 to 332050), only 3% reduction relative to no self-isolation (Table 1). For total hospital beds, this level of self-isolation leads to the requirement for 2,737,924 (IQR 2,139,967 to 3,271,920) beds at outbreak peak, a 9.8% reduction compared to no isolation. The outbreak peak would be delayed by at most 1 wk.

**Table 1.** The projected peak capacity requirements and time to peak for hospitalized non-ICU and ICU patients, across a range of self-isolation scenarios

	1/τ = 2 d				1/τ = 1 d			
	Required capacity at peak (IQR)		Time to peak (weeks)		Required capacity at peak (IQR)		Time to peak (weeks)	
*f*	Non-ICU	ICU	Non-ICU	ICU	Non-ICU	ICU	Non-ICU	ICU
*R*_0_ = 2.5								
5%	2,479,076 (1,972,872–2,940,206)	284,688 (236,860–332,052)	15	16	2,225,577 (2,042,651–2,562,605)	230,427 (188,988–279,898)	18	18
10%	2,453,629 (2,304,903–2,672,102)	264,152 (237,375–300,564)	18	19	1,862,475 (1,537,488–2,225,491)	205,393 (168,517–244,433)	20	21
20%	2,091,018 (1,905,777–2,382,647)	221,641 (191,376–264,698)	20	21	1,378,390 (1,219,462–1,618,818)	151,028 (133,803–172,753)	27	28
*R*_0_ = 2								
5%	1,293,506 (1,210,773–1,438,487)	132,554 (116,068–152,427)	22	22	1,020,660 (935,319–1,187,839)	105,103 (89,458–125,157)	26	26
10%	1,164,271 (1,052,032–1,347,699)	120,454 (102,735–140,603)	23	23	753,001 (642,169–867,162)	76,844 (65,310–89,307)	35	35
20%	975,796 (855,538–1,139,402)	104,033 (88,716–121,693)	27	28	390,756 (348,111–454,936)	40,128 (34,153–47,440)	56	57

Reported estimates are mean and IQR. *f*: proportion of individuals with mild symptoms who practice self-isolation. 1/τ: average time to self-isolation postsymptom onset for individuals with mild symptoms.

Accelerating self-isolation or increasing the proportion of cases who self-isolate would further reduce cumulative hospitalizations and ICU admissions per 1,000 population across all age groups (Fig. 1 *A* and *D*). Moreover, changes in the speed and proportion of self-isolation have a significant impact on the peak capacity requirements, as well as time to peak (Table 1). If 20% of mild cases self-isolate 48 h after symptom onset, the projected peak ICU bed requirement would be reduced by 24.6% (IQR 22.5 to 26.7%) and delayed by 4 wk, relative to no self-isolation (Table 1). The benefits of self-isolation are substantially increased when initiated earlier in the disease course. For example, when 20% of mildly symptomatic individuals practice self-isolation at 24 h, the peak weekly requirement for ICU beds would be reduced by 48.4% (IQR 46.4 to 50.3%) and delayed by 12 wk (Fig. 1 *C* and *F*), relative to no self-isolation. This requirement corresponds to 4.4 times the typically available ICU beds. Expanding self-isolation at 24 h to 85% of mildly symptomatic individuals would drop the peak requirement for ICU beds to the typically available capacity.

We observed qualitatively similar trends for *R*_0_ = 2 (Fig. 2), with rapid self-isolation suppressing and delaying the peak incidence of hospitalizations (Table 1). The proportional impact of any given measure is substantially higher when *R*_0_ = 2. For example, when self-isolation was initiated at 24 h by 20% of mildly symptomatic individuals, the peak demand for ICU beds was reduced by 73.5% (IQR 71.4 to 75.3%) and delayed by up to 38 wk, relative to no self-isolation. In this scenario, 1.2 times the available ICU beds would be required at the peak. To ensure that peak ICU requirements would not exceed available capacity, at least 24% of mildly symptomatic individuals would need to self-isolate within 24 h of symptom onset.

## Discussion

As an increasing number of cases are being identified due to community spread of SARS–CoV-2 in the United States, it is imperative to evaluate the effectiveness of control measures on reducing disease burden and inform decisions on optimal implementation of intervention strategies. Adequate ICU capacity is crucial to save the lives of severe COVID-19 cases. Our projections indicate that COVID-19 will overwhelm hospital capacity in the United States at the peak of the outbreak. A similar demand was experienced during the initial wave of the 2009 H1N1 pandemic, as well as the 2003 SARS epidemic in some population settings (10). In these instances, the critical care facilities were compromised by the surge of cases.

Consistent with advice from the CDC, our results indicate that early identification of symptomatic cases combined with timely self-isolation can dramatically reduce the demand for hospital and ICU beds. However, self-isolation alone is unlikely to be sufficient for keeping peak ICU demand below the typically available capacity. With an *R*_0_ of 2.5, we estimated that about 85% of mildly symptomatic individuals would need to self-isolate within 24 h to avoid exceeding typically available critical care capacity. Such an expectation may not be realistic and is highly dependent on the means provided to make it possible to remain at home.

The timelines of self-isolation are more influential than the proportion of mildly symptomatic individuals who self-isolate. For example, our results show that 20% of symptomatic individuals with mild illness self-isolating 48 h after symptom onset resulted in comparable outcomes to 5% of them self-isolating 24 h after symptom onset. Given that 80% of all COVID-19 cases have been classified as mild (11, 12), yet likely spreading the disease, timely self-isolation as well as behavioral avoidance should be considered a key public health consideration (13). This is particularly important, since individuals with mild illness taking symptom-suppressing drugs may reduce their rates of self-isolation, thereby contributing to the spread of disease further. Moreover, timely diagnosis and self-isolation can delay the peak-time of outbreak and hospitalization between 5 and 30 wk, providing the time for additional resources to be rolled out.

Our study raises several analytical and public policy challenges beyond the scope of this paper. We have focused on the role of self-isolation to slow the epidemic and enable the healthcare system to manage critical care of symptomatic patients and continue non–COVID-19 healthcare activities. Yet self-isolation should be seen within the broader framework of nonpharmaceutical interventions to reduce infectious contacts, including a range of social-distancing policies. Our model does not incorporate social-distancing policies other than self-isolation for lack of adequate parametrization of their effects, yet there could well be important synergies between self-isolation and other social-distancing policies. In the current crisis, China has deployed a range of social-distancing policies (14), and globally, school closures have been widely adopted [in 13 countries affecting 292 million school children as of March 4, 2020 (15)]. In the context of respiratory infections (e.g., influenza), school closure has been shown to reduce visits to the emergency department at the hospitals and slow the progression of the outbreak (16, 17). However, assessing the effectiveness of school closures is challenging, as it may depend on the population setting and the time of implementation (16). Limiting public transportation would also reduce the number of contacts with infectious cases, particularly those with mild symptoms, and therefore impede the spatial spread of the disease. However, this approach requires careful evaluation before implementation as it may indirectly affect access to healthcare and other essential services (18, 19).

Self-isolation can also play a major role in preventing local outbreaks in long-term care facilities by minimizing disease importation through daily visitors who may be infected with mild illness (20). Residents of long-term care facilities are particularly vulnerable to communicable diseases (e.g., influenza, COVID-19) due to underlying health conditions and possible congregation during daily activities among themselves and with visitors (20, 21). A recent COVID-19 outbreak in a nursing home in Washington State, causing several deaths, highlights the stark vulnerability of these facilities (21, 22).

Given the potentially critical role of self-isolation, it is also extremely important to consider the policy framework for promoting effective self-isolation. This might include some or all of the following policies: Public awareness campaigns to educate the public on the reasons for self-isolation and public support for those in self-isolation, including call-in numbers for notification and information; use of telemedicine sites that essentially make consultation about conditions available to everyone; access to testing at home for COVID-19 infection; home visits by trained community health workers and social workers; home delivery of medicines for symptomatic relief; home provision of other support (such as hygienic materials to reduce transmission within households); guaranteed paid sick leave; flexible work-from-home policies; and perhaps fines or other sanctions for flagrantly negligent behavior by symptomatic individuals in spreading the infection.

The results of this study should be considered within the context of model assumptions. Our results are based on early estimates of the parameters relying on limited amounts of data from initial outbreaks in China. As these estimates are refined, our model can be reparameterized to provide more accurate projections. We also assumed that hospitalized patients are effectively isolated and do not transmit the disease to others (e.g., healthcare workers), thereby making hospitalization a perfect isolation setting. Our projections for critical care needs are conservative in two regards. First, we only considered symptomatic transmission in our model. Under the possibility of asymptomatic and presymptomatic transmission (23), self-isolation of only symptomatic cases would be much less effective in mitigating the need for hospitalization and ICU beds. Second, our results correspond to the overall projections at a national level. Geographic disparities and other spatially heterogeneous factors among the US states and counties, combined with stochasticity, would likely exacerbate the imbalance between supply and demand more than we project here. ICU beds may go unused in locations with no or mild outbreak while hotspots are overwhelmed.

Our findings highlight that the available hospital and ICU beds would be overburdened by needs of critically ill patients at the peak of the COVID-19 outbreak in the United States. In addition to underscoring the urgency of ICU capacity expansion, our results suggest that prosocial self-isolation will delay the timing and alleviate the extent of the COVID-19 surge, thereby mitigating the strain on the healthcare system.

## Methods

### Transmission Dynamics

We modeled the transmission of SARS–CoV-2 using a compartment-based system of differential equations (*SI Appendix*, Fig. A1). The model stratified the US population into four age groups: 0 to 19, 20 to 49, 50 to 64, and 65+ y of age, parameterized from United States census data (24). These age groups were chosen based on availability of disease-specific parameters, and also to provide results that are aligned with the age stratification often used for other respiratory infections like influenza (25). Transmission of SARS–CoV-2 depended on an empirically determined age-specific contact matrix for communities and households (26). Newly infected individuals entered an incubation period for an average of 5.2 d before becoming symptomatic (*SI Appendix*, Table A5). Combining with the estimated average of 7.5 d for the serial interval (27), we calculated an average infectious period of 4.6 d, during which individuals were concurrently symptomatic. Symptomatic cases had an age-dependent probability of developing mild, severe, or critical illness. We assumed that patients exhibiting mild symptoms recover without hospitalization, while severe and critical cases had an age-dependent probability of non-ICU and ICU hospital admissions, respectively. We also assumed that the relative infectivity of mild illness compared to severe and critical illness is reduced by 50% (28).

For those individuals who become hospitalized, the average time from onset of symptoms to hospital admission was randomly sampled in the range 2 to 5 d (9). During the time prior to admission, these individuals were considered to be as infectious as individuals who were not ultimately hospitalized. Upon admission, hospitalized patients are effectively isolated and no longer contribute to community transmission. Severe (non-ICU patients) and critical (ICU patients) cases recovered following an average of 10 and 13.25 d of hospital stay, respectively (29) (*SI Appendix*, Table A5). Based on initial epidemiological evidence, we assumed that 23.5% of all hospitalized cases die (9). The average time from admission to death for non-ICU and ICU patients was 9.7 and 7 d, respectively (9, 30).

### Self-Isolation

Self-isolation was defined as limiting contacts to household members during symptomatic infection and avoiding contact with the broader community. We assumed that all individuals with severe or critical illness would self-isolate either immediately following symptom onset (5%) or within 1 d of symptom onset (80%). Among the 80% of infected individuals with mild symptoms (31), we considered self-isolation of individuals after symptom onset in the range 5 to 20%, and evaluated its impact on the hospitalization rates and need during COVID-19 outbreaks. Symptomatic individuals who practiced delayed self-isolation did so within 24 or 48 h of symptom onset, depending on the scenario.

### Model Calibration

We calibrated the SARS–CoV-2 transmission parameters to initial estimates (9) of the reproduction number for *R*_0 _= 2.5 for the base case, and *R*_0 _= 2 as an alternative scenario. Other parameter values were informed by estimates of SARS–CoV-2 disease characteristics and were sampled from relevant distributions when available (*SI Appendix*, Table A5). All simulations were seeded with one initial symptomatic case in each age group, and the results were averaged over 100 independent realizations.

### Data Availability

The computational system is available at https://github.com/affans/ncov2019odemodel.

## Supplementary Material

Supplementary File
