# Supplementary material for: Projecting hospital utilization during the COVID-19 outbreaks in the United States
Source: Proc Natl Acad Sci U S A. 2020 Apr 3;117(16):9122–6. doi: 10.1073/pnas.2004064117 (PMC7183199; doi:10.1073/pnas.2004064117)
Supplement: Supplementary File [file pnas.2004064117.sapp.pdf]

Appendix

Classification: Biological Sciences, Population Biology

Projecting hospital utilization during the COVID-19 outbreaks in the United States

Authors: Seyed M. Moghadas,[1] Affan Shoukat,[2] Meagan C. Fitzpatrick,[3] Chad R Wells,[2] Pratha Sah,[2] Abhishek Pandey[2], Jeffrey D. Sachs,[4] Zheng Wang,[5] Lauren A. Meyers[6] , Burton H Singer,[7] Alison P. Galvani[2]

[1]Agent-Based Modelling Laboratory, York University, Toronto, Ontario, M3J 1P3 Canada
[2]Center for Infectious Disease Modeling and Analysis (CIDMA), Yale School of Public Health, New Haven, Connecticut, USA
[3]Center for Vaccine Development and Global Health, University of Maryland School of Medicine, 685 W Baltimore St, Baltimore, MD 21201, USA
[4]Center for Sustainable Development at Columbia University, Columbia University, New York, NY 10032, USA
[5]Department of Biostatistics, Yale School of Public Health, New Haven, Connecticut, USA
[6]The University of Texas at Austin, Austin, Texas
[7]Emerging Pathogens Institute, University of Florida, Gainesville, FL 32610, USA

Corresponding author: *bhsinger@epi.ufl.edu*

Keywords: SARS-CoV-2, hospitalization, surge capacity, self-isolation

Model

Transmission dynamics
We developed an age-structured dynamic transmission model to simulate the spread of the novel coronavirus (SARS-CoV-2) in the United States (US). We divided our population study into four () age groups in the model, including 0-19, 20-49, 50-64, and 65+ years of age. Infection occurs through age-dependent contacts between susceptible and infectious (symptomatic) individuals, defined by a contact matrix M *within the community, and a contact matrix* $\tilde{M}$ *within households. Once susceptibles (*S*) become infected, they follow the natural history of the disease, and remain in the incubation period (*E*) for an average period of* $1/\sigma$ *days. After the incubation period has elapsed, infected individuals develop symptomatic disease. A proportion* θ *of these*

symptomatic cases develop a mild form of illness, while the remaining exhibit more severe and critical illness.

For the severe and critical cases, we assumed a proportion q *of patients immediately self-isolate themselves (based on the knowledge of having contacts with infectious cases) within their home (*Q*) and the remaining proportion continues to follow normal activity in the general population (*I*). Of these severe cases who did not self-isolate directly upon symptom onset, a fraction* f_I *will practice self-isolation after diagnosis during symptomatic disease (corresponding to an average of* $1/\tau_I$ *days after symptom onset).*

A proportion, $1-h$*, of severe and critical symptomatic patients will recover without the need for hospitalization or critical care. The remaining proportion will require hospitalization (and/or intensive care in ICU). Patients not needing hospitalized care experience an average* $1/\gamma$ *duration from onset of symptoms to recovery. For patients requiring hospitalization (*H*), the average time from symptom onset to hospital admission is* $1/\delta$ *days. A fraction* c *of admitted patients will occupy ICU beds (*C*).*

We assumed that a proportion m_H *of hospitalized patients die after an average period of* $1/\mu_H$ *days post admission, while the remaining proportion recover with an average* $1/\psi_H$ *length of hospital stay. Similarly, for those admitted to the ICU, a proportion* m_C *die after an average duration of* $1/\mu_C$ *days post admission. The remaining fraction* $1-m_C$ *of ICU patients recover after an average length of* $1/\psi_C$ *days.*

For the cases experiencing mild illness (A*), we assumed that they do not immediately self-isolate upon symptom onset. Rather, a proportion* f_A *of these mild cases practice self-isolation after* $1/\tau_A$ *days from symptom onset (a parameter that was varied in our simulations). In addition, these cases exhibiting mild symptoms do not require hospitalization or admission to the ICU. We also assumed that the relative infectivity of mild illness compared to severe and critical illness is reduced by 50%.*

The dynamics of infection and control measures described here are schematically illustrated in Figure A1, and presented by equations (A1)-(A10) in the model. This model was used to investigate the effect of timely identification of symptomatic cases, self-isolation, and determine the hospital surge capacity required for treatment of severely and critically ill patients.

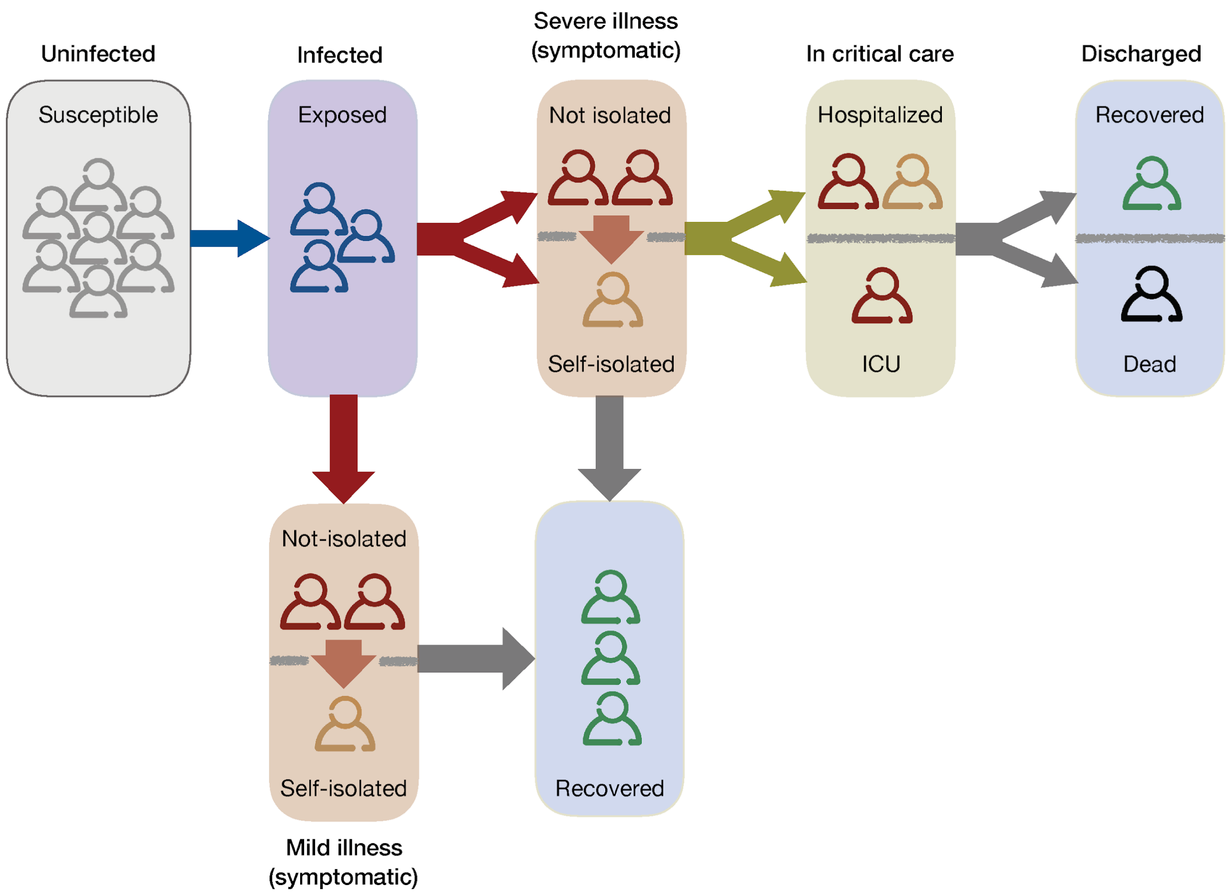

Figure A1: *Schematic diagram of the model for natural history of the disease and implementation of self-isolation and hospitalization.*

Table A1: *Description of the compartments for the COVID-19 transmission model*

Variable	Description
S_a	Susceptibles in age group a
E_a	Exposed individuals in age group a
$A_{N,a}$	Mild symptomatic cases (without self-isolation) who will not need hospitalization in age group a
$A_{Q,a}$	Mild symptomatic cases who practice self-isolation and will not need hospitalization in age group a
$I_{N,a}$	Severe symptomatic cases (without self-isolation) who will not need hospitalization in age group a
$Q_{N,a}$	Severe symptomatic cases who practice self-isolation and will not need hospitalization in age group a
$I_{H,a}$	Severe or critical symptomatic cases (without self-isolation) who will need hospitalization in age group a
$Q_{H,a}$	Severe or critical symptomatic cases who practice self-isolation and will need hospitalization in age group a
H_a	Number of cases in the hospital (non-ICU) for age group a
C_a	Number of cases in the ICU for age group a
P_a	Population size for age group a.

System of equations

$$\frac{dS_a}{dt} = -\beta S_a \sum_{j=1}^{\lambda} M_{a,j} \frac{(I_{N,j}+I_{H,j}+\kappa A_{N,j})}{P_j} - \beta S_a \sum_{j=1}^{\lambda} \tilde{M}_{a,j} \frac{(Q_{N,j}+Q_{H,j}+\kappa A_{Q,j})}{P_j} \quad \text{(A1)}$$

$$\frac{dE_a}{dt} = \beta S_a \sum_{j=1}^{\lambda} M_{a,j} \frac{(I_{N,j}+I_{H,j}+\kappa A_{N,j})}{P_j} + \beta S_a \sum_{j=1}^{\lambda} \tilde{M}_{a,j} \frac{(Q_{N,j}+Q_{H,j}+\kappa A_{Q,j})}{P_j} - \sigma E_a \quad \text{(A2)}$$

$$\frac{dI_{N,a}}{dt} = (1-\theta_a)(1-q)(1-h_a)\sigma E_a - (1-f_I)\gamma I_{N,a} - f_I \tau_I I_{N,a} \quad \text{(A3)}$$

$$\frac{dQ_{N,a}}{dt} = (1-\theta_a)q(1-h_a)\sigma E_a - \gamma Q_{N,a} + f_I \tau_I I_{N,a} \quad \text{(A4)}$$

$$\frac{dI_{H,a}}{dt} = (1-\theta_a)(1-q)h_a \sigma E_a - (1-f_I)\delta I_{H,a} - f_I \tau_I I_{H,a} \quad \text{(A5)}$$

$$\frac{dQ_{H,a}}{dt} = (1-\theta_a)q h_a \sigma E_a - \delta Q_{H,a} + f_I \tau_I I_{H,a} \quad \text{(A6)}$$

$$\frac{dA_{N,a}}{dt} = \theta_a \sigma E_a - (1-f_A)\gamma A_{N,a} - f_A \tau_A A_{N,a} \quad \text{(A7)}$$

$$\frac{dA_{Q,a}}{dt} = f_A \tau_A A_{N,a} - \gamma A_{Q,a} \quad \text{(A8)}$$

$$\frac{dH_a}{dt} = (1-c_a)(1-f_I)\delta I_{H,a} + (1-c_a)\delta Q_{H,a} - (m_H \mu_H + (1-m_H)\psi_H) H_a \quad \text{(A9)}$$

$$\frac{dC_a}{dt} = c_a(1-f_I)\delta I_{H,a} + c_a \delta Q_{H,a} - (m_C \mu + (1-m_C)\psi_C) C_a \quad \text{(A10)}$$

Table A2: *Description of the parameters included in the system of equations*

Parameter	Description	Units
β	Transmission rate	per contact
$M_{a,c}$	Contact rate between people in age group *a* and people in age group *c*	contacts per day
$\tilde{M}_{a,c}$	Household contact rate between people in age group *a* and people in age group *c*	contacts per day
κ	Relative infectivity of mild illness compared to severe and critical illness	
$1/\sigma$	Average duration of incubation period	days
$1/\tau_I$	Average time from symptom onset to self-isolation for severe and critical cases	day
$1/\tau_A$	Average time from symptom onset to self-isolation for mild cases	day
θ	Proportion of cases that exhibit mild symptoms	
q	Proportion of severe symptomatic cases who practice self-isolation upon symptom onset	
h	Proportion of symptomatic patients with severe and critical illness requiring hospital care or ICU	
c	Proportion of hospitalized cases requiring ICU	
f_I	Weight for self-isolation after symptom onset for severe and critical cases	
f_A	Weight for self-isolation after symptom onset for mild cases	
$1/\gamma$	Average time from symptom onset to recovery	days
$1/\delta$	Average time from symptom onset to hospitalization	days
$1\mu_C$	Time from ICU admission to death	day
$1\mu_H$	Time from hospitalization (non-ICU) admission to death	day
m_C	Weight associated with the model death rate among ICU patients	
m_H	Weight associated with the model death rate among hospitalized (non-ICU) patients	
$1/\psi_H$	Length of hospital stay before recovery	days
$1/\psi_C$	Length of ICU stay before recovery	days
λ	The number of age groups	

Description of parameters and their estimation

Mortality
We define the probability of mortality in the hospital to be p_m*. The mortality rate is denoted by* μ*, the rate to recovery in the hospital is denoted by* ψ*, and model weight of mortality in the hospital is denoted by* m*. Therefore, the probability of mortality in the hospital is expressed as*

$$p_m = \frac{m\mu}{m\mu + (1-m)\psi}. \tag{A11}$$

Thus, the weight assigned to the mortality rate is

$$m = \frac{p_m\psi}{p_m\psi + (1-p_m)\mu}. \tag{A12}$$

Contact Matrix

We used an estimated contact structure for the US that was based on contact surveys and demographic data (1). This structure was based on age classes spanning five years, ranging from 0-4 to 75-79. We first aggregated the number of contacts for each of our age classes 0-19, 20-49, 50-64, and 65+, with the age stratification of contacts still at five year intervals. Since the contact structure went to age 79, we used the span of 65-79 demographics for the purpose of compressing the contact matrix from the demographic data (2). We then determined the average number of contacts for in individual class j *based on our age stratification and the demographics of the US:*

$$X_{i,j} = \frac{\sum_{n\le a_j} N_n K_{n,i}}{\sum_{n\le a_j} N_n}, \tag{A13}$$

where N_n *is the number of individuals in age range* $n-4$ *to* n*,* a_j *is the maximum age for class* j*,* $K_{n,j}$ *is the aggregated number of contacts in age rage* $n-4$ *to* n *for age class* i*. We then determined the symmetric contract matrix by evaluating the average number of contacts*

$$M = \frac{(X + X^T)}{2}. \tag{A14}$$

For non-isolated individuals, we used the contact matrix that specified all locations, while the home contact matrix was utilized for isolated individuals (1).

Table A3: *The community contact matrix for the various age groups*

M	**0-19**	**20-49**	**50-64**	**65+**
**0-19**	9.76	3.77	1.51	0.60
**20-49**	3.77	9.43	3.05	0.70
**50-64**	1.51	3.05	2.96	0.76
**65+**	0.60	0.70	0.76	1.25

Table A4: *The isolation contact matrix for the various age groups*

$\tilde{M}$	**0-19**	**20-49**	**50-64**	**65+**
**0-19**	2.04	1.56	0.50	0.38
**20-49**	1.56	1.51	0.45	0.24
**50-64**	0.50	0.45	1.04	0.19
**65+**	0.38	0.24	0.19	0.64

Average length of stay in ICU
We used the average time spent on a ventilator as a measure for the length of stay in the ICU. The median time spent on an invasive ventilator was 17 days, where cases spent a median of nine days on non-invasive ventilation (3). Of the cases in the ICU, 47.2% were on invasive ventilation and 41.7% required noninvasive ventilation (4). Normalizing based on ventilation only, the average time spent on ventilation is approximately 13.25 days.

Infectious period (used as a proxy for time to recovery from onset of symptoms in non-hospitalized patients)

We used the average duration of the serial interval (7.5 days) and the average duration of the incubation period (5.2 days), to approximate the infectious period of cases that would not be hospitalized (5). Since infection is random, the infectious period is twice the difference of the average serial interval and average incubation

period. Thus, we estimate the infectious period to be 4.6 days.

Calibration of transmission rate for Reproductive Number
To calibrate the transmission parameter β for R_0=2 and 2.5, we used the next generation method (5, 6). For calibration, we considered that there is no self-isolation ($q = 0$ and $f = 0$).

Table A5: *Description of parameters and their values/ranges.*

**Description (units in days)**	**Variable**	**0-19**	**20-49**	**50-64**	**65+**	**Reference**
Transmission rate	β	0.0493, 0.0616	0.0493, 0.0616	0.0493, 0.0616	0.0493, 0.0616	Calibrated to R_0=2, 2.5
Relative infectivity of mild illness compared to severe and critical illness	κ	0.5	0.5	0.5	0.5	(7)
Average duration of incubation period	$1/\sigma$	LogN (5.2, 0.1)	LogN (5.2, 0.1)	LogN (5.2, 0.1)	LogN (5.2, 0.1)	(5)
Proportion of cases that exhibit mild symptoms (based on reported cases in different age groups)	θ	0.80	0.80	0.40	0.20	Assumed
Proportion of severe symptomatic cases who practice self-isolation upon symptom onset	q	0.05	0.05	0.05	0.05	Assumed
Average infectious period	1/©	4.6	4.6	4.6	4.6	Estimated
Weight for self-isolation after symptom onset for severe and critical cases	f_I	0.80	0.80	0.80	0.80	Assumed
Weight for self-isolation after symptom onset for mild cases	f_A	0.05 - 0.2	0.05 - 0.2	0.05 - 0.2	0.05 - 0.2	Varied

Average time from symptom onset to self-isolation for severe and critical cases	$1/\tau_I$	1	1	1	1	Assumed
Average time from symptom onset to self-isolation for mild cases	$1/\tau_A$	1-2	1-2	1-2	1-2	Varied
Average time from symptom onset to hospitalization	$1/\delta$	Unif(2,5)	Unif(2,5)	Unif(2,5)	Unif(2,5)	(8)
Proportion of symptomatic patients with severe and critical illness requiring hospital care or ICU	h	0.025	0.32	0.32	0.64	Estimated from (8, 9)
Proportion of hospitalized cases requiring ICU	c	0.014	0.042	0.075	0.15	Estimated from(8)
Proportion of hospitalized cases that die	p_m	0.235	0.235	0.235	0.235	Average(8)
Time from hospitalization (non-ICU) admission to death	$1/\mu_H$	9.7	9.7	9.7	9.7	Average (8)
Length of hospital stay before recovery	$1/\psi_H$	10	10	10	10	(4)
Weight associated with the model death rate among hospitalized (non-ICU) patients	m_H	0.2296	0.2296	0.2296	0.2296	Calculated
Time from ICU admission to death	$1/\mu_C$	7	7	7	7	(10)
Length of ICU stay before recovery	$1/\psi_C$	13.25	13.25	13.25	13.25	Estimated
Weight associated with the model death rate among ICU patients	m_C	0.1396	0.1396	0.1396	0.1396	Calculated

Population size (2018)	*P*	81982665	129596376	63157200	52431193	(2)

Projected disease-induced death rate

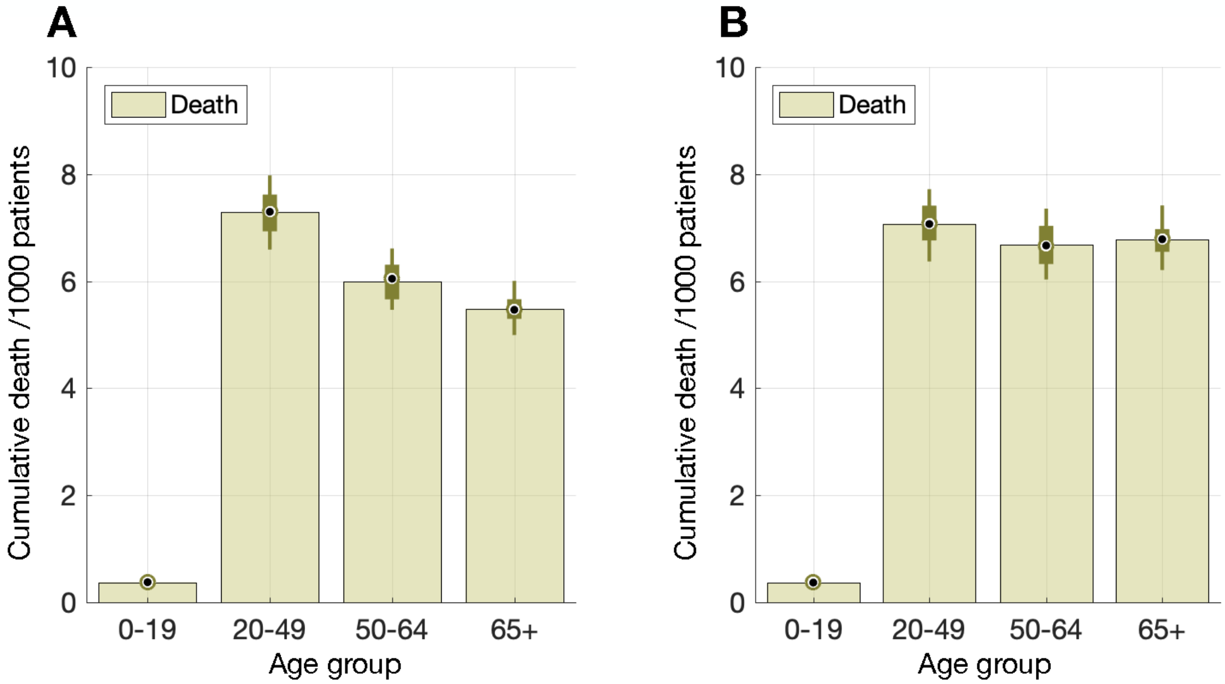

Figure A2. *Projected rates of death per 1000 patients in different age groups with (A) R_0=2, and (B) R_0=2.5. Scenarios presented here correspond to 5% self-isolation one day after symptom onset. Colour bars illustrate the mean values, and box plots indicate the median and interquartile range (IQR) of estimates.*

Results for the incidence of Hospitalization at the outbreak peak

Table A6: *The projected number and time to peak of hospitalization for weekly incidence of all non-ICU and ICU patients for 5%, 10%, and 20% self-isolation of individuals with mild illness. Reported estimates are mean and interquartile range (IQR) for an average time of two days and one day to self-isolation after symptom onset.*

		$1/\tau$ = 2 days						$1/\tau$ = 1 day				
		Weekly incidence at peak (IQR)			Time to peak (weeks)			Weekly incidence at peak (IQR)			Time to peak (weeks)	
R_0=2.5	*f*	Non-ICU	ICU		non-ICU	ICU		Non-ICU	ICU		non-ICU	ICU
	5%	1,977,792 (1,756,289 – 2,247,367)	187,528 (163,955 - 212592)		15	16		1,660,505 (1,510,103 – 1,913,314)	15,2381 (130,440 – 181,238)		18	18
	10%	1,606,428 (1,399,439 – 1,862,190)	148,162 (127,084 – 172,038)		18	19		1,452,042 (1,281,246 – 1,644,254)	132,968 (112,203 – 155,489)		20	21
	20%	1,452,042 (1,281,246 – 1,644,254)	132,968 (112,203 – 155,489)		20	21		1,020,907 (925,355 – 1,183,133)	93,175 (83,664 – 107,196)		27	28
R_0=2												
	5%	937,883 (871,737 – 1,027,012)	83,779 (74,701 – 94,936)		22	22		740,437 (662,009 – 832,803)	66,031 (57,640 – 75,584)		26	26
	10%	846,694 (763,526 – 969,444)	76,104 (67,171 – 86,179)		23	23		541,545 (485,132 – 620,644)	47,500 (39,704 – 54,664)		35	35
	20%	714,651 (655,716 – 825,268)	63,633 (56,010 – 73,053)		27	28		277,665 (249,308 – 322,613)	23,963 (20,116 – 28,554)		56	57

f : proportion of individuals with mild symptoms who practice self-isolation
1/τ: average time to self-isolation post symptom onset for individuals with mild symptoms
ICU: intensive care unit

References

1. *K. Prem, A. R. Cook, M. Jit, Projecting social contact matrices in 152 countries using contact surveys and demographic data. PLoS Comput. Biol.* ***13****, e1005697 (2017).*

2. , *"Annual Estimates of the Resident Population by Single Year of Age and Sex for the United States: April 1, 2010 to July 1, 2018 (PEPSYASEXN)" (US Census Bureau, 2019) (February 29, 2020).*

3. *N. Chen, et al., Epidemiological and clinical characteristics of 99 cases of 2019 novel coronavirus pneumonia in Wuhan, China: a descriptive study. The Lancet* ***395****, 507–513 (2020).*

4. *D. Wang, et al., Clinical Characteristics of 138 Hospitalized Patients With 2019 Novel Coronavirus–Infected Pneumonia in Wuhan, China. JAMA (2020) https:/doi.org/10.1001/jama.2020.1585.*

5. *Q. Li, et al., Early Transmission Dynamics in Wuhan, China, of Novel Coronavirus-Infected Pneumonia. N. Engl. J. Med. (2020) https:/doi.org/10.1056/NEJMoa2001316.*

6. *J. T. Wu, K. Leung, G. M. Leung, Nowcasting and forecasting the potential domestic and international spread of the 2019-nCoV outbreak originating in Wuhan, China: a modelling study. The Lancet* ***395****, 689–697 (2020).*

7. *R. Li, et al., Substantial undocumented infection facilitates the rapid dissemination of novel coronavirus (SARS-CoV2). Science (2020) https:/doi.org/10.1126/science.abb3221.*

8. *midas-network, midas-network/COVID-19. GitHub (February 27, 2020).*

9. , *"Report of the WHO-China Joint Mission on Coronavirus Disease 2019 (COVID-19)" (World Health Organization, 16-24 February 2020) (March 9, 2020).*

10. *X. Yang, et al., Clinical course and outcomes of critically ill patients with SARS-CoV-2 pneumonia in Wuhan, China: a single-centered, retrospective, observational study. The Lancet Respiratory Medicine (2020) https:/doi.org/10.1016/s2213-2600(20)30079-5.*
